# Supplementary material for: Content-rich biological network constructed by mining PubMed abstracts
Source: BMC Bioinformatics. 2004 Oct 8;5:147. doi: 10.1186/1471-2105-5-147 (PMC528731; doi:10.1186/1471-2105-5-147)
Supplement: Additional File 2 — The original results of the above study (non-essential files are deleted to keep the file size under the limit set by BMC bioinformatics). [file 1471-2105-5-147-S2.bz2 › chilibotAdditionalFile2/dip05/401ID7539796E9997/html/CNTF_CNTFR.html]

 


 **CNTF** and **CNTFR** 
  
Found 97 abstracts in PubMed, retrieved 05.  
 

 What does Google say? 
 PDF only 
| .edu only 

---

**Interactive relationship** (e.g. stimulation, inhibition, etc)

**Inhibitory relationship**- Human  **CNTF**  elicits cellular responses by induction of a receptor complex consisting of the  **CNTF**  alpha receptor  **CNTFR** , which is not involved in signal transduction, and the beta receptors gp130 and leukemia inhibitory factor receptor LIFR.  Ref: 12643274 J Biol Chem, 2003
- The functional receptor complex of ciliary neurotrophic factor  [ **CNTF** ]   **CNTF** , a member of the gp130 family of cytokines, is composed of  **CNTF** , the  **CNTF**  receptor alpha  **CNTFR** , gp130 and the leukemia inhibitory factor receptor LIFR.  Ref: 12707266 J Biol Chem< MedlineTA>J Biol Chem, 2003
**Neutral relationship**- Engineering a  **CNTFR**  specific human  **CNTF**  variant may therefore be a prerequisite to improving the safety profile of  **CNTF** .  Ref: 12643274 J Biol Chem, 2003
- These data suggest that both the gp130 CBD and the LIFR CBD1 interact with  **CNTFR**  in a similar way and provide insights into the nature of the  **CNTF**  receptor complex.  Ref: 12707266 J Biol Chem< MedlineTA>J Biol Chem, 2003

**Non-interactive relationship** (e.g. studied together, co-existance, homology, etc.)

- We performed gene screening of the ciliary neurotrophic factor  [ **CNTF** ]  receptor  **CNTFR**  gene and genotyped three newly identified polymorphisms C 1703T in the 5 promoter region, T1069A in intron 5, and C174T in exon 9.  Ref: 12807897 J Appl Physiol< MedlineTA>J Appl Physiol, 2003
- The C174T polymorphism in the  **CNTF**  receptor  **CNTFR**  gene is associated with fat free mass in men and women.  Ref: 12807897 J Appl Physiol< MedlineTA>J Appl Physiol, 2003
- We previously reported that the membrane distal cytokine binding domain CBD1 of LIFR could interact in vitro with soluble  **CNTFR**  sCNTFR in the absence of  **CNTF** .  Ref: 12707266 J Biol Chem< MedlineTA>J Biol Chem, 2003
